# Topical Nano and Microemulsions for Skin Delivery

**DOI:** 10.3390/pharmaceutics9040037

**Published:** 2017-09-21

**Authors:** Christofori M. R. R. Nastiti, Thellie Ponto, Eman Abd, Jeffrey E. Grice, Heather A. E. Benson, Michael S. Roberts

**Affiliations:** 1 School of Pharmacy, Curtin Health Innovation Research Institute, Curtin University, G.P.O. Box U1987, Perth, WA 6845, Australia; c.nastiti@postgrad.curtin.edu.au (C.M.R.R.N.); thellie.ponto@postgrad.curtin.edu.au (T.P.); H.Benson@curtin.edu.au (H.A.E.B.); 2 Faculty of Pharmacy, Sanata Dharma University, Yogyakarta 55282, Indonesia; 3 Therapeutics Research Centre, The University of Queensland Diamentina Institute, Faculty of Medicine, Translational Research Institute, Woolloongabba, QLD 4102, Australia; e.abd@uq.edu.au (E.A.); jeff.grice@uq.edu.au (J.E.G.); 4 School of Pharmacy and Medical Sciences, University of South Australia, Adelaide, SA 5000, Australia

**Keywords:** microemulsion, nanoemulsion, transdermal, skin penetration, penetration enhancer, nanosystem

## Abstract

Nanosystems such as microemulsions (ME) and nanoemulsions (NE) offer considerable opportunities for targeted drug delivery to and via the skin. ME and NE are stable colloidal systems composed of oil and water, stabilised by a mixture of surfactants and cosurfactants, that have received particular interest as topical skin delivery systems. There is considerable scope to manipulate the formulation components and characteristics to achieve optimal bioavailability and minimal skin irritancy. This includes the incorporation of established chemical penetration enhancers to fluidize the stratum corneum lipid bilayers, thus reducing the primary skin barrier and increasing permeation. This review discusses nanosystems with utility in skin delivery and focuses on the composition and characterization of ME and NE for topical and transdermal delivery. The mechanism of skin delivery across the stratum corneum and via hair follicles is reviewed with particular focus on the influence of formulation.

## 1. Introduction

**Figure 1:**
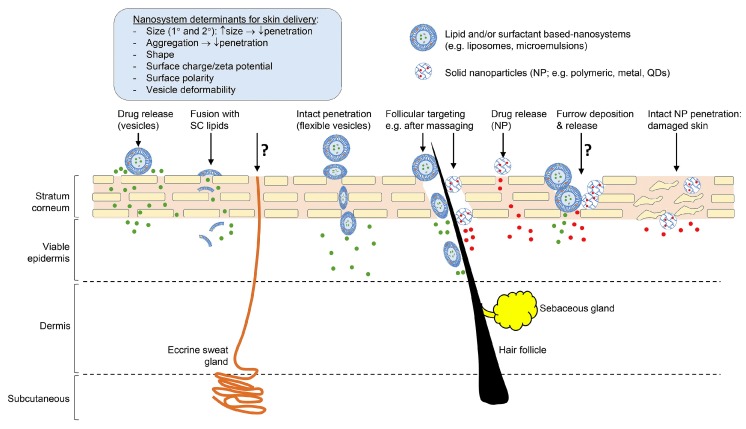
Properties of nanosystems determining skin absorption and potential routes of penetration (skin layer thicknesses not drawn to scale). Reproduced with permission from [5].

The skin provides an effective barrier to protect the body from the penetration of molecules and micro-organisms in the external environment, and from excessive loss of water to maintain homeostasis. The main skin barrier resides in the stratum corneum (Figure 1) due to its unique structure of layers of flattened corneocytes surrounded by lipid bilayers composed primarily of ceramides [1]. Penetration of most topically applied compounds follows the tortuous route of the stratum corneum lipid bilayers (intercellular) [2], although the transcellular route through the corneocytes may contribute in some circumstances [3]. Although hair follicles (and associated sebaceous glands) and sweat glands account for only about 0.1% of the total skin surface area [4], these appendages are potential routes of access into the skin, and may be important for nanosystems.

Compounds that successfully diffuse across the stratum corneum are typically relatively small (up to about 500 Da), lipophilic (logP 1–3) and water soluble, thus excluding many potentially useful therapeutic compounds with properties that do not fit these criteria. A range of micro- and nanosystems has been investigated as potential delivery vehicles that could enhance the skin penetration of both small and macromolecules that do not otherwise permeate the stratum corneum in sufficient quantities to provide a therapeutic outcome. Here, we review micro and nanosystems that have been applied to skin delivery and focus particularly on micro- and nanoemulsions, as these present an extension of the most commonly applied topical formulations used in pharmaceutical, cosmeceutical and personal care products.

## 2. Classification of Nano and Microsystems Used for Skin Delivery

Nanosystems that have been investigated for enhanced skin permeation include microemulsions (ME), nanoemulsions (NE), nanoparticles of various compositions including solid lipid nanoparticles (SLN), nanostructured lipid carriers (NLC), liposomes and vesicles [5]. These nanosystems can offer significant advantages in the formulation of hydrophobic molecules, enhancing their solubility and thus bioavailability. This approach has been used to formulate hydrophobic actives for a range of routes of administration, including topical application to the skin. An example is Estrasorb^®^ (Novavax Inc., Malvern, PA, USA), which contains oestradiol hemihydrate (logP 3.3) in a nanoemulsion composed of soybean oil, water, polysorbate 80 and ethanol, packed in single dose foil pouches for application to the legs in the management of vasomotor symptoms associated with menopause. Topicaine^®^ (ESBA Laboratories Inc., Jupiter, FL, USA) is a microemulsion-based gel product (composed of jojoba oil, aloe vera oil, ethanol, benzyl alcohol, glycerine and water emulsified by glyceryl monostearate, and gelled with carbomer 940) containing lidocaine for localised pain relief. There are also examples of nanosystems designed to enhance delivery of hydrophilic compounds. Ameluz^®^ topical gel (Biofrontera Pharma GmbH, Leverkusen, Germany) containing aminolevulinic acid (logP 1.5) in a nanoemulsion composed of soybean phosphatidylcholine, water, polysorbate, propylene glycol and isopropyl alcohol for the treatment of actinic keratosis and basal cell carcinoma. The range of nanosystems used for skin delivery is classified below. Figure 1 summarises the suggested and/or established mechanisms of skin permeation of nanosystems. While some routes are well described in the literature, the extent to which other routes, such as the eccrine sweat glands and via skin furrows (designated as ? in Figure 1), contribute to skin permeation is less well established. We have demonstrated that topically applied zinc oxide nanoparticles deposit in the skin furrows, but this does not contribute to permeation to deeper skin tissues [6,7].

*Microemulsions (ME):* transparent, monophasic, optically isotropic and thermodynamically stable colloidal dispersions composed of oil, water, surfactant and cosurfactant with droplet sizes in the range 10–100 nm [8].

*Nanoemulsions (NE):* transparent, monophasic, optically isotropic and kinetically stable colloidal dispersions composed of oil, water, surfactant and cosurfactant with droplet sizes less than 100 nm.

*Solid Nanoparticles*: Discrete particles in the size range up to 1000 nm composed of inorganic materials such as metal oxides (e.g., zinc oxide, titanium dioxide) or polymers. There is a considerable body of evidence to show that these nanoparticles do not permeate human skin under a range of administration conditions [6,7]. Their primary application is as sunscreen products.

*Solid Lipid Nanoparticles (SLN):* composed of lipids that are solid at room temperature with a surface covering of surfactant to stabilise them as a nano-dispersion [9]. SLN enhance skin permeation by prolonging contact with the skin surface, providing an occlusive barrier that hydrates the skin, and interacting with the lipids in the stratum corneum bilayers. They are particularly useful for formulation of hydrophobic actives such as vitamin A, E and coenzyme Q in cosmetically elegant products, and maintaining the stability of compounds such as retinol that are prone to decomposition by light and oxygen [9].

*Nanostructured lipid carriers (NLC):* colloid systems composed of a fluid lipid phase embedded into a solid lipid matrix or localized at the surface of solid platelets and the surfactant layer [9,10]. The spatial structure of the lipids allows greater drug loading and better stability compared to SLN.

*Liposomes:* spherical vesicles composed of amphiphilic phospholipids and cholesterol, self-associated into multilamellar, large unilamellar and small unilamellar vesicles.

*Flexible vesicles:* composed of materials that will associate into bilayer structures but incorporate components that confer flexibility, thereby allowing the vesicles to deform in shape. Compositions that associate into flexible vesicles include ethosomes (phospholipids with a high proportion of ethanol) [11], niosomes (non-ionic surfactants) [12], invasomes (phospholipids, ethanol and a mixture of terpene penetration enhancers) [13], SECosomes (surfactant, ethanol and cholesterol) [14] and PEV (penetration enhancer vesicles, for which a range of penetration enhancers have been investigated including oleic acid, limonene, propylene glycol, Transcutol^®^) [15,16]. Multiple mechanisms are likely to contribute to enhanced skin permeation including the effect of the vesicle components on the stratum corneum lipids and the potential, as proposed by Cevc, that the vesicles have sufficient flexibility to squeeze through the stratum corneum intact [17,18].

*Polymeric micelles and dendrimers*: nanosized, colloidal carriers with a hydrophilic exterior shell and a hydrophobic interior core, comprised of two main categories of hydrophobically assembled micelles and polyion-complex micelles [19]. Dendrimers are highly branched polymer structures incorporating drug and potentially, penetration enhancer molecules [20].

## 3. Formulation of Micro and Nanoemulsions

The focus of this review is the application of ME and NE in dermal and transdermal drug delivery. In comparison to many of the nanosystems outlined above, ME and NE offer advantages in terms of simplicity and stability. Coarse emulsions are composed of oil and water phases, with one dispersed as droplets in the other, and stabilised by a surfactant. In addition to their obvious droplet size difference, ME are clear/transparent, form spontaneously, have low interfacial energy and are thermodynamically stable, unlike emulsions that are cloudy, require energy in preparation, have high interfacial energy and are kinetically stable [21]. While the terminology suggests that NE would have a smaller particle size than ME, based on nano and micro referring to 10^−9^ and 10^−6^ respectively, this is a quirk of the history of the development of colloidal dispersions, and the size range of NE and ME is similar. Essentially, the terms ME and NE entered widespread usage before they were properly defined or distinguished from each other [22]. Both NE and ME typically have low polydispersity (up to about 10%) compared to the much higher polydispersity exhibited by emulsions (>40%).

NE are thermodynamically unstable but kinetically stable, and can be prepared by both low and high energy methods. Given sufficient time, an NE will phase separate. Destabilization mechanisms include flocculation, coalescence, Ostwald ripening and creaming, with Ostwald ripening being the dominant mechanism of destabilization for NE [22,23,24]. The systems also differ when exposed to dilution and temperature fluctuations. ME are affected and potentially broken by temperature changes and/or dilution, whereas NE droplets will remain stable under these physical stresses [23]. Thus, the primary difference between NE and ME is their thermodynamic stability [22], which also results in the higher energy input required to form NE compared to ME. Detailed examination of the terminology, differences and similarities of NE and ME, with particular focus on their physical chemistry, is provided by McClements [22], Anton and Vandamme [23] and Gupta et al. [24]. The similarities and differences between emulsions, ME and NE are summarized in Table 1.

The low interfacial tension and small particle size in ME and NE is due to their composition; in particular, the presence of cosurfactants such as short or medium chain alcohols or polyglyceryl derivatives working in combination with the primary surfactant [25]. The surfactant to oil ratio is much higher in ME (see examples in Table 2) than in coarse emulsions (typically 2–10%). While coarse emulsions are creamy in appearance and tend to adhere well to the skin, NE and ME are more fluid. To achieve an appropriate consistency of ME or NE on the skin, a viscosity enhancing polymer is added to form a gel.

In general, non-ionic surfactants are favoured as they are less irritating to human skin. A wide range of non-ionic surfactants and the amphiphilic surfactant lecithin have been investigated, together with a variety of oils and cosurfactants (Table 2). The development of NE and ME formulations is based on ternary diagrams to determine the optimal component ratios.

## 4. Formulation Parameters: Composition and Preparation Methods

ME can be formed spontaneously at optimal component ratios and temperature, although in practice, low energy such as heat or stirring is generally applied to facilitate formation. ME and NE are classified as water in oil (w/o) or oil in water (o/w), each designating the dispersed phase within the continuous phase. Some more complex systems also exist such as o/w/o and w/o/w. Bicontinuous ME in which the aqueous and oil phases are intertwined and stabilized by sheet-like surfactant areas in the areas between the phases [26] can also exist. Lindman et al. [27] demonstrated that continuous pathways can exist between interconnected-sphere structures, lamellar-like structures, tubule structures and other structures in ME systems. Bicontinuous structures are dynamic and are characterized by a higher amphiphilic character, greater fluctuation at the interface, lower interfacial tension and better solubilizing properties compared to globular w/o or o/w ME [28,29]. Naoui et al. [29] compared the penetration of the hydrophilic drug caffeine across excised pig skin, when applied in o/w, w/o and bicontinuous ME having the same ingredients. Transdermal flux of caffeine was in the order w/o < bicontinuous < o/w ME, with the o/w ME providing permeation of 50% of the applied dose within 24 h. In contrast, Bhatia et al. [28] reported that, for the lipophilic drug adapalene, the penetration in hair follicles increased by almost three times as the microstructure of the applied ME shifted from o/w to bicontinuous, with an increase in water content of the ME. In both cases, presentation of the drug in the continuous phase of the ME provided the greatest drug delivery, with the bicontinuous system acting as an intermediate system.

### 4.1. Preparation Methods

**Figure 2:**
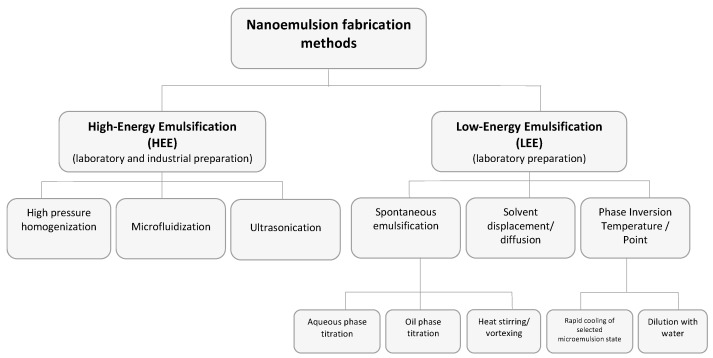
Schematic representation of nanoemulsion preparation methods adapted from [30,31].

NE formation is generally a two-step process with the initial preparation of a macroemulsion that is then converted to a NE. This requires external energy applied by high-energy (HEE) or low-energy (LEE) methods (Figure 2). HEE methods such as high-pressure homogenizers, microfluidizers and ultrasonicators generate highly disruptive forces that break down the oil and water phases, causing them to intersperse and form nanometer-sized droplets. LEE methods include heat, stirring and phase inversion. Control of NE droplet size is related to both the preparation method and the formulation components.

Dilution can be used as a method of forming a final ME or NE product. NE can be prepared by diluting o/w microemulsions, bicontinuous microemulsions, or w/o ME with water [32]. Dilution of an o/w ME with water induces a proportion of the surfactants to dissolve into the aqueous phase. The surfactant molecules remaining at the oil/water interface cannot maintain the low interfacial tension required for thermodynamic stability and the ME droplets give rise to nanoemulsion droplets [33].

When diluting bicontinuous ME, the homogeneous nucleation that occurs during the spontaneous emulsification process leads to the formation of NE [34] Despite this mechanism, NE may be formed by the migration of surfactants or cosurfactants through the oil-water interface due to the “ouzo effect” when diluting bicontinuous ME or w/o ME [35]. When diluting w/o ME, the oil may act as nuclei, leading to heterogeneous nucleation, resulting in droplets with larger sizes and polydispersity [36]. NE formed by diluting o/w ME or bicontinuous ME are more stable and have smaller droplets [37].

Sole et al. [32] reported that NE with droplet diameters of 20 nm were obtained when diluting o/w ME regardless of the ME composition or dilution procedure (incremental or all at once). In contrast, when the starting emulsion was a w/o ME, NE were only obtained if the emulsification conditions allowed the establishment of an equilibrium in an o/w ME domain during the process. These conditions required the stepwise addition of water and w/o ME with specific oil to surfactant ratios.

### 4.2. Composition

The choice of emulsion components and ratios of these components is critical in generating stable emulsion systems with appropriate particle sizes. A wide range of components and combinations has been investigated (Table 2).

Oil phase components include fatty acids (e.g., oleic acid), esters of fatty acids and alcohols (e.g., isopropyl myristate, isopropyl palmitate, ethyl oleate), medium chain triglycerides, triacetin, terpenes (e.g., limonene, menthol, cineole) and other penetration enhancers. These may be used alone or in combination to form the oil phase. The aqueous phase may include sodium chloride and buffer salts, preservatives and penetration enhancers. Viscosity enhancing agents (e.g., Carbopol^®^, Aerosil^®^, gelatin) are incorporated to reduce the fluidity and generate the desired final consistency of the product.

A wide range of materials has been used as surfactants and cosurfactants (see examples in Table 2). Consideration must be given to combinations that effectively reduce interfacial tension and produce stable emulsions with appropriate particle size, but which also ensure minimal skin irritancy; thus, the preference for non-ionic surfactants. Commonly used surfactants include Tween^®^ (polysorbates), Cremophor^®^ (mixture of macrogol glycerol hydroxystearate, PEG-40 castor oil, polyoxyl 40 hydrogenated castor oil), Transcutol^®^ P (diethylene glycol monoethyl ether), Plurol Oleique^®^ (polyglyceryl-3-oleate), Plurol Isostearique^®^ (isostearic acid ester of poly-glycerols and higher oligomers) and Labrasol^®^ (mixture of mono-, di- and tri-glycerides of C8 and C10 fatty acids, and mono- and di-esters of PEG) [38]. Lecithin, an amphiphilic compound, has been widely investigated as the “ideal” surfactant because it is a natural compound with a low skin irritancy profile. Organogels are w/o ME based on lecithin and an apolar organic solvent, that form gel-like reverse micellar systems with high viscosity, solubilisation capacity and thermodynamic stability, and are transparent and biocompatible [39]. Cosurfactants are generally short and medium chain alcohols and polyglyceryl derivatives, including ethanol, isopropanol, isopropyl myristate and propylene glycol (PG). Nonionic surfactants have also been used to provide low irritancy cosurfactants [38,40]. Table 2 shows examples of the range of NE and ME compositions and their skin delivery. Lopes provides an excellent review focused on the formulation and physical characterisation of ME [41].

## 5. Physical Characterisation of Nano- and Microemulsions

### 5.1. Pseudo Ternary Phase Diagrams

**Figure 3:**
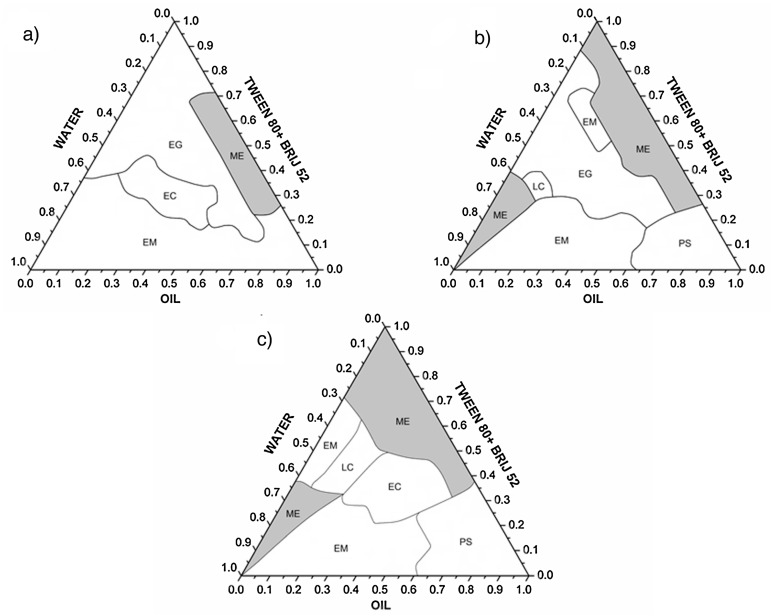
Pseudo-ternary phase diagrams formed by a mixture of caprylic/capric triglycerides as the oil phase, Tween 80: Brij 52 at 7:3 (**a**), 8:2 (**b**) and 9:1 (**c**) surfactant mix-ratio and water. Gray area represents the microemulsion systems (ME). Liquid crystal (LC), emulsion (EM), emollient gel (EG), emollient cream (EC), phase separation (PS). Reproduced with permission from [43].

NE and ME are characterised by a range of physical properties that are important determinants of their structure, drug release and stability. Pseudo ternary phase diagrams are often constructed to indicate the boundaries of the different phases as a function of the composition of the aqueous, oil and surfactant/cosurfactant components [21,42]. Mixtures of the oil, surfactant and cosurfactant at certain weight ratios at ambient temperature (25 °C) are diluted with the aqueous solution under moderate agitation. After equilibrium, the combinations of the three components that give rise to clear emulsions, shown by visual inspection or polarised light microscopy, are mapped on the phase diagram. Examples of pseudo-ternary phase diagrams showing regions of various phases for mixtures of oil and water and different ratios of surfactant and cosurfactant (Tween 80 and Brij 52) are shown in Figure 3 [43].

### 5.2. Particle Size, Polydispersity and Zeta Potential

Particle/droplet size and polydispersity index can be determined by microscopic and scattering techniques. Dynamic light scattering, also called photon correlation spectroscopy (PCS), is used to analyse the fluctuations in intensity of incident laser light as it passes through droplets or particles that are subject to Brownian motion. A number of instruments are available that can provide rapid analysis of the particle/droplet size (down to about 1 nm), polydispersity (a measure of the broadness of the size distribution derived from the cumulative analysis of dynamic light scattering: indicates the quality or homogeneity of the dispersion) and zeta potential (surface charge).

Freeze fracture transmission electron microscopy (TEM) and cryo-TEM allow the direct imaging of nanostructures at high resolution [44]. Laser light scattering, photon correlation spectroscopy (PCS), small angle X-ray scattering (SAXS), small angle neutron scattering (SANS) are useful for determining particle size distribution [45]. Consideration of particle interactions within the emulsion systems, and understanding the limitations of these techniques, is critical to ensure accurate measurements [21].

### 5.3. Viscosity and Electrical Conductivity

Viscosity and conductivity measurements provide information on the emulsion structure and can be used to detect phase inversion phenomena [45]. High conductivity values demonstrate a water continuous phase, whilst an oil continuous phase will have low or no conductivity. Where the conductivity increases this may demonstrate the percolation effect caused by the attractive interactions between water droplets, that is characteristic of a bicontinuous structure [46,47]. These measurements can also be useful in predicting drug release from the NE or ME.

Electrical conductivity meaurements are simple and inexpensive, involving the insertion of conductometer electodes into the NE/ME formulation. High conductivity is obtained for an aqueous continuous phase, and phase inversion in response to formulation or temperature change can be monitored by the change in electrical conductivity. The technique is therefore useful for determining emulsion type, and monitoring changes during preparation or storage [48].

Viscosity is an important property that influences the stability and drug release of NE and ME formulations. The viscosity of an emulsion is a function of the surfactant, water and oil components and their concentrations. An increase in the water content will lead to a lowering of the viscosity, while decreasing the surfactant and co surfactant content increases interfacial tension between the water and oil, causing increased viscosity. Monitoring of viscosity changes is a method of assessing the stability of liquid and semi-solid preparations, including nanoemulsion formulations [49]. In general, cone and plate type rheometers are used for rheological evaluation of NE and ME [50].

Podlogar et al. [45] provide an excellent example of how the data from a range of these techniques can be collectively interpreted to provide the structural characterisation of ME. They found good agreement in their measurements of density and surface tension, and by viscometry, conductivity, DSC and SAXS techniques, for their ME (composed of IPM and water with Tween 40^®^ and Imwitor 308^®^ [glyceryl caprylate] surfactant/cosurfactant mix). The SAXS data showed a monodisperse w/o ME with strong attractive interactions. The type of ME was confirmed by DSC by demonstrating the degree of interaction between water and surfactants. Conductivity, viscosity, density and surface tension measurements confirmed a percolation transition to a bicontinuous structure. The authors concluded that these techniques could be applied to determine the type and structure of more complex systems, and could enable partitioning and release rates of drugs from ME to be predicted.

## 6. Skin Delivery from Nano and Microemulsions

NE and ME systems have been developed for the delivery of a wide range of compounds to the skin for dermatological, cosmetic/cosmeceutical and transdermal outcomes. Enhanced skin delivery has been demonstrated in comparison to conventional emulsions and gels. This has been attributed both to the action of their components on the skin and their phase structure and particle size. We have evaluated the literature on in vitro and in vivo studies of skin permeation of compounds applied as NE and ME systems, with particular focus on the formulation composition and properties, and skin permeation experimental design. The choice of appropriate models for skin permeation evaluation is critical to the accurate assessment of the potential of these systems as future therapeutic products. Ideally, studies are conducted on human excised skin or volunteers, although pig and piglet skin does provide a reasonable surrogate. In studies of follicular penetration, Lademann has suggested that pig ear skin is a superior in vitro model, as it does not contract and close the follicle openings, as excised human skin does [51]. Animal models such as rat, mouse and rabbit have a weaker barrier than human skin and their use tends to over-estimate skin permeation relative to humans. In addition, experimental parameters such as the appropriate choice of receptor solutions that do not damage skin membranes, while providing sufficient receptor phase solubility to achieve sink conditions and suitable hydrodynamics to limit the formation of aqueous diffusion layers [52] need to be scrutinized, along with validated analytical methods and application protocols. In some cases high proportions of alcohols [53,54,55,56,57] or other known penetration enhancers such as DMSO [58] have been used to provide sink conditions in the receptor phase, with the potential to compromise the skin barrier and lead to over-estimation of drug flux. Our work has also shown that even when non-sink conditions are used in in vitro permeation experiments, the results can be corrected to derive the equivalent sink condition data, provided the effects of aqueous diffusion layers are minimised [52].

Given the presence of sebum in hair follicles it is likely that oil, surfactant and alcohol based vehicles such as NE/ME could facilitate transfollicular transport of both hydrophilic and lipophilic compounds. Bhatia et al. [28] indicated that ME not only increased the permeation of adapalene in the stratum corneum, but also demonstrated optimal penetration into the hair follicles. The permeation of adapalene in the stratum corneum increased from 1.40 to 3.37 μg and penetration in the hair follicles increased significantly from 0.017 to 0.292 μg in ME treated skin compared with the control. This represents a 17-fold increase in penetration in the hair follicles compared with the control. Teichmann et al. [59] compared the skin penetration of the lipophilic dye curcumin incorporated in an o/w ME and a coarse emulsion/cream applied to human volunteers. Using the method of tape stripping to remove the stratum corneum (SC), the depth profiles of the dye within the horny layer were compared. The depth of penetration, determined both by tape stripping and laser scanning microscopy, was greater with the ME than the cream. In addition, when applied in the ME, curcumin penetrated into the complete follicular infundibula, whereas with the cream a fluorescence signal was only received from the follicular orifices.

While some hair follicles are open, others are plugged with shed corneocytes and dry sebum [60], which can particularly restrict the permeation of hydrophilic compounds. Hair follicles can be opened by a mechanical peeling technique applied prior to the administration of a topical formulation [61]. We investigated the follicular delivery of the hydrophilic compound caffeine from NE composed of penetration enhancer chemicals (unpublished data). We found that when we open the hair follicles, the increase in caffeine permeation relative to control (aqueous solution) was greater for oleic acid and eucalyptol NE. The cumulative amount and flux of caffeine increased by 27- and 23-fold with oleic acid NE relative to control. Eucalyptol NE increased the cumulative amount of caffeine penetrated by 43-fold and flux by 31-fold compared to control.

In the following section, we discuss representative literature on a range of formulations, focused on the anti-inflammatory drug class. Examples of NE formulations evaluated for a broad range of therapeutic classes relevant to topical and transdermal delivery are also summarised in Table 2.

### Anti-Inflammatory Drugs

Non-steroidal anti-inflammatory drugs (NSAIDs) are widely used in the management of musculoskeletal and arthritic pain. These drugs often create gastro-intestinal side effects when taken orally, thus application to the skin over the painful site is an attractive alternative. A number of NSAIDs have been available as gel and cream formulations for many years. There is extensive literature focused on the development and evaluation of ME and NE systems for topical delivery of a number of NSAIDs including diclofenac [62,63], aceclofenac [57,64,65], piroxicam [66], indomethacin [67,68,69], ibuprofen, celecoxib [70,71], etoricoxib [72], naproxen [73], flufenamic acid [50,74,75], ketoprofen [39,76,77], flurbiprofen [78], lornoxicam [79], and meloxicam [80]. Consequently, we have focused our discussion on this drug class to illustrate the development and potential of ME/NE formulations for topical and transdermal delivery.

ME composed of oleic acid as the internal phase, Labrasol^®^/Cremophor^®^ RH as the cosolvent mixture and water were shown to enhance skin permeation of the lipophilic NSAID ketoprofen [76]. Increasing the water content (from 5 to 64%) and reducing the surfactant content (from 80 to 30%) increased ketoprofen skin permeation. This increased permeation is achieved by reducing the solubility of the drug and hence increasing its thermodynamic activity in the external phase, and has been reported for other lipophilic compounds [74,81]. Hoppel et al. [74] evaluated a lecithin related, naturally derived monoacyl phosphatidycholine (MAPL) surfactant, with the aim of reducing the irritancy associated with many conventional ionic surfactants. The in vitro skin permeation of flufenamic acid across dermatomed porcine skin was evaluated from ME composed of oleic acid, water, MAPL and isopropanol as co-surfactant. Attenuated total reflectance–fourier transform infrared spectrometry (ATR-FTIR) analysis and tape stripping of the stratum corneum demonstrated that the MAPL itself did not penetrate beyond the superficial layers of the stratum corneum. This superficial penetration is likely to minimise irritancy. NSAID skin permeation was significantly greater for the water-rich ME than other ME compositions and a commercial flufenamic acid product. When applied to the skin the isopropanol evaporated, leaving crystal-like structures of MAPL on the skin surface and forming a barrier to skin permeation. However, the ME with high water content prevented the formation of these MAPL structures. In a subsequent study exploring the influence of MAPL content it was confirmed that higher MAPL content resulted in lower skin permeation of flufenamic acid, most likely due to the MAPL acting as a hydrophilic barrier to the permeation of the lipophilic drug [50].

Duangjit et al. [82] applied a simple lattice statistical design approach to provide a more rational choice of ME composition. The physical properties (size, charge, conductivity, pH, viscosity, drug content and loading capacity) and skin permeation were determined for ketoprofen-loaded ME composed of oleic acid, Cremophor^®^ RH, ethanol and water. The authors reported that the experimentally determined skin permeation correlated well with their predictions using Design-Expert^®^ software (Stat-ease, Minneapolis, MN, USA), and allowed optimisation of skin delivery via rational design.

Oleic acid-based ME have also been investigated for skin delivery of flufenamic acid [74,75,83]. Mahrhauser et al. [75] combined a fluorosurfactant (Hexafor^TM^670 or Chemguard S-550-100) with isopropyl alcohol as cosurfactant (total S + CoS 65% *w*/*w*) to form an anisotropic ME with oleic acid (10% *w*/*w*) and water (25% *w*/*w*), loaded with flurbiprofen. Physical characterisation using conductivity, SAXS and NMR showed that the ME was oil in water with spherical or rod-shaped microstructures. In vitro porcine skin penetration demonstrated enhanced permeation for the ME with elongated rather than spherical microstructures, suggesting that the shape of the ME particles is an important determinant in skin delivery. In a parallel study incorporating diclofenac sodium in the ME, increased deposition into the stratum corneum of porcine ear skin was demonstrated by tape stripping [83]. ATR-FTIR studies showed significant shifts of the CH_2_ stretching absorbance when the ME was applied to the skin, demonstrating increased disorder of the stratum corneum lipids that was indicative of reduced barrier function. Similar shifts were not seen when pure fluorosurfactant was applied, suggesting that the permeation enhancement was a feature of the ME and not simply the surfactant constituent.

Sugar-based esters have been investigated as another source of low irritancy surfactants [84]. Sucrose esters [laurate (SL) or myristate (SM)] were shown to be superior surfactants to Tween^®^ 80 (T80) for the delivery of aceclofenac from ME composed of isopropyl myristate, water and co-surfactant of isopropyl alcohol or Transcutol^®^P. Aceclofenac release from the ME was determined across cellulose membranes and in vivo tape stripping (12 strips) was performed on human volunteers. An in vivo pharmacokinetic study was conducted in rats and skin irritancy of blank ME determined on human volunteers by measuring trans epidermal water loss (TEWL), erythema and hydration. The ME incorporating sugar esters released significantly more aceclofenac over 6h than Tween 80-based ME (87.28 ± 4.89 and 70.66 ± 4.46 compared to 53.65 ± 5.62% for SL, SM and T80 respectively). Aceclofenac penetration into the stratum corneum (by tape stripping) from the sugar ester ME was approximately two times that of the T80 ME (60.81 ± 5.97, 60.86 ± 3.67, 27.00 ± 5.09 mg/cm^2^ for SL, SM, T80 respectively), and this was reflected in the maximum plasma concentrations and lag times measured in the rats (275.57 ± 109.49, 281.32 ± 6.76, 150.23 ± 69.74 ng/mL and 0.44 ± 0.19, 0.74 ± 0.32, 2.41 ± 2.70 h for SL, SM, T80 respectively). Not only were the sugar ester-based ME effective in the transdermal delivery of aceclofenac, but they also showed better skin tolerability [84].

Kriwet and Müller–Goyman [85] explored the mechanism of lecithin-based permeation enhancement by altering the ratio of diclofenac diethylamine, lecithin (soybean phosphatidylcholine) and water to develop a range of colloidal structures including ME, liposomes and lamellar liquid crystals. As diclofenac diethylamine is an amphiphilic molecule it interacts with the colloidal microstructure. At lecithin concentrations below 6% low viscosity ME were formed which gave rapid release of diclofenac diethylamine across a silicone impregnated dialysis membrane. Increasing the lecithin concentration led to phase transition into isotropic gels containing droplets with few lamellar layers surrounding the droplets. The increased viscosity of these systems resulted in decreased drug release. ME also gave higher permeation of diclofenac across human stratum corneum membranes than the other formulations tested, including a simple aqueous solution. The authors suggested that the increased permeation resulted from interaction between the lecithin phospholipids and the stratum corneum lipid bilayers, but this occurred only when presented as a ME and not as gel or liposomal formulations. In the liposomal formulations, the drug and phospholipids are too tightly held within the colloidal microstructure to effectively interact with the stratum corneum [85]. Further investigation of the interaction of lecithin-based ME and the stratum corneum was undertaken using FTIR and differential scanning calorimetry (DSC) [86]. In this case they used isopropyl palmitate as the oil phase (alone and in the lecithin-based ME) and investigated the in vitro permeation of indomethacin and diclofenac across full-thickness human skin and the interactions on isolated stratum corneum sheets. ME formulations provided much higher permeation compared to isopropyl palmitate solutions, for both drugs. ME and isopropyl palmitate alone gave similar temperature shifts of the stratum corneum lipid transitions so they could not distinguish the penetration enhancement role of lecithin.

Viscosity enhancing agents are often added to convert the ME into a gel consistency suitable for retention on the skin surface. Naeem et al. [87] compared gels composed of Carbopol^®^ 934P and Xanthan gum bases containing ME (5% *w*/*w* oleic acid, 46% *w*/*w* Tween^®^20:ethanol 2:1, 44% *w*/*w* water) or hydroalcoholic solution (ethanol/water) incorporating 5% *w*/*w* flurbiprofen. The transdermal flux of flurbiprofen across excised rabbit skin was 18.75 ± 0.08, 15.72 ± 0.05, 9.80 ± 0.09, 4.76 ± 0.07 and 2.70 ± 0.05 μg/cm^2^/h over 24 h for the un-gelled ME, ME gelled with Carbopol and Xanthan, and hydroalcoholic solution gelled with Carbopol and Xanthan respectively. This clearly demonstrates that the ME delivers more NSAID than the hydroalcoholic solutions and that addition of a gelling agent reduces transdermal delivery. Similar findings were reported for lornoxicam [79] and aceclofenac [57], although it is interesting to note that this group have reported identical transdermal flux values for the two NSAIDs in these separately published manuscripts, despite the different drugs, compositions and experimental models. Shakeel et al. [67] also reported lower transdermal flux of indomethacin across rat skin from ME composed of 5% Labrafil^®^, 50% water and 45% of a 3:1 ratio of Tween^®^ 80 and Transcutol^®^, when gelled with 1% Carbopol^®^ 940 (73.96 ± 2.89 and 61.64 ± 2.38 μg/cm^2^/h). It should be noted that there are other reports in which the addition of gelling agent did not significantly change the skin delivery (e.g., diclofenac diethylamine [63]) or indeed resulted in an increase in transdermal flux (e.g., amphotericin [58]).

The incorporation of additional chemical penetration enhancers [dimethyl sulfoxide (DMSO) and propylene glycol (PG)] in w/o ME compositions has been shown to further increase skin permeation across excised rabbit skin [88]. The relative effects of DMSO and PG were shown to be dependent on the cosurfactant in the ME formulation. PG gave better skin permeation of diclofenac sodium than DMSO when incorporated into isopropyl alcohol ME, whereas DMSO was superior to PG in propanol ME. Overall the ME containing isopropyl alcohol and PG gave greatest enhancement, although all ME formulations provided higher skin permeation of diclofenac than the commercial products tested.

We examined the skin permeation enhancement of the lipophilic NSAID naproxen and the hydrophilic drug caffeine applied in NE incorporating skin penetration enhancers oleic acid or eucalyptol as oil phases, with Volpo-N10 (an ethoxylated fatty alcohol) and ethanol as the surfactant and cosurfactant in a 1:1 ratio [73]. Caffeine and naproxen fluxes across human epidermal membranes were determined over 8 h. All NE formulations significantly enhanced the skin penetration of both caffeine and naproxen, compared to aqueous control solutions. Caffeine maximum flux enhancement was associated with a synergistic increase in both caffeine stratum corneum solubility and skin diffusivity, whereas a formulation-increased solubility in the stratum corneum was the dominant mechanism for increased naproxen fluxes. Enhancements in stratum corneum solubility were related to the uptake into the stratum corneum of the formulation excipients containing the active compounds. We concluded that enhanced skin penetration from NE is primarily due to uptake of formulation excipients containing the active compounds into the stratum corneum with consequent impacts on stratum corneum solubility and diffusivity.

A number of in vivo pharmacokinetic studies have supported the enhanced skin delivery effects of ME demonstrated in vitro. For example, an eight-fold higher permeation of diclofenac from ME (w/o; 2:3 PEG-40 stearate/glyceryl oleate as surfactant mix, tetraglycol as cosurfactant, S/CoS ratio 8:1, isopropyl myristate as oil phase and water than Voltaren^®^ Emulgel (commercial coarse emulsion gel product) was demonstrated in a rat pharmacokinetic study [89]. Constant plasma diclofenac levels of 0.7–0.9 μg/mL were maintained for at least 8 h following ME administration. In contrast, a subcutaneous injection of diclofenac solution (3.5 mg/kg) resulted in a peak plasma level of 0.94 μg/mL at 1 h, which decreased rapidly to 0.19 μg/mL by 6 h.

Ensuring that any novel formulation maintains the stability and therapeutic activity of the drug is essential. The anti-inflammatory activity of NSAIDs applied in ME formulations has been demonstrated in a number of studies. For example, ME [o/w: composed of isopropyl myristate, water, Capmul MCM^®^ (mixture of medium chain glycerides), Tween 80], gel (added Carbopol^®^ 934), and cream (anionic emulsifying ointment and water) formulations containing celecoxib were compared for permeation across excised full-thickness rat skin [70]. Selected formulations were evaluated using the arachidonic acid induced ear edema model in Swiss albino mice. The skin permeation of celecoxib from the ME formulations was 3 to 5-fold greater than ME gels, and 7 to 11-fold greater than the cream. Increasing the concentration of Capmul MCM^®^ in the ME resulted in an increase of droplet size and viscosity and decrease in celecoxib diffusion coefficient. Administration of selected ME formulations reduced ear edema by up to 55% demonstrating that the celecoxib ME was an effective anti-inflammatory formulation.

## 7. Conclusions and Future Directions

ME and NE have a clear place in the delivery of active compounds to and through the skin for a range of therapeutic purposes. They are elegant, relatively simple and inexpensive to manufacture and offer significant delivery advantages over coarse emulsions. Over the past few decades, there has been extensive research demonstrating the effectiveness of these delivery technologies. In addition, the development of new excipients with potential utility in NE and ME formulations continues to offer new opportunities for formulations with high delivery capacity coupled with low irritancy and toxicity. Although it has been explored, the precise mechanism of delivery of these formulations remains controversial, but it is likely to be a combination of the effect of the formulation components on stratum corneum diffusivity of the active compounds. The increase in transfollicular penetration from NE and ME is well established and is again likely due to both the formulation components facilitating penetration through the sebum and within the follicle. Given the advantages of these systems and the continued development of low toxicity excipients, it is likely that we will continue to see new NE and ME products for topical and transdermal delivery into the future.

## Figures and Tables

**Table 1:** Comparison of the properties of emulsions, microemulsions and nanoemulsions.

	Emulsion	Microemulsion	Nanoemulsion
Physical description	Coarse dispersion	Colloidal dispersion	Colloidal dispersion
Particle size range	>500 nm	<100 nm	<100 nm
Polydispersity	High	Low	Low
Thermodynamic stability	Unstable	Stable	Unstable
Preparation	High energy	Low energy	Low/high energy
Composition: surfactant to oil ratio	Low	High	Moderate
Physical appearance	Creamy	Transparent	Transparent
Texture	Semi-solid	Fluid	Fluid

**Table 2:** Examples of nanoemulsion (NE) formulations evaluated for topical and transdermal delivery: hydrophilic (H) and lipophilic (L) nature of active compound, composition (detail where available), preparation method and physical characterisation of emulsion formulation, and skin permeation experimental details and data.

Therapeutic Class and Active Compound	H/L	Composition	Preparation Method	Physical Characterisation				Skin Permeation Evaluation		Ref
				Particle Size (nm)	Surface Charge (mV)	Poly Dispersity	Viscosity (mPa s)			
**NON-STEROIDAL ANTI-INFLAMMATORY DRUGS (NSAID)**										
Aceclofenac	L	Nanoemulsion NE31 (O/W) Triacetin 13.6% Water 54.6% Cremophor EL^®^ 23.9% PEG-400 7.9% Nanoemulsion gel NG31 NE31 gelled with Carbopol 934^®^ 1% Drug load (DL): 1.5 mg%	Spontaneous aqueous phase titration	NE31 39.48		NE31 0.230	NE31 339.51 ± 0.31	Method (M)	Full thickness rat abdominal skin Receptor: methanol-PBS (pH 7.4) (3:7)	[57]
								Results (R)	*Flux J (μg·cm^−2^·h^−1^) in 24 h* NE31: 254.90 ± 1.25 NG31: 199.60 ± 6.93 *Control* (Hiffenac™ Gel) 43.67 ± 2.11 *Enhancement ratio* (*ER*) NE31: 5.84 NG31: 4.57	
Aceclofenac (ACF)	L	Nanoemulsion L_1.5_ S_0.5_ P_2_A Medium chain triglycerides (MCT) Castor oil 1:1 20% Water 76% Lecithin 80 1.5% Sucrose stearate S-970 0.5% Sucrose palmitate P-1670 2% Drug load (DL): 1% *w*/*w*	High pressure homogenization	181.2 ± 0.8	−39.2 ± 1.5	0.110 ± 0.006	3.60 ± 0.23	(M)	Human skin (in vivo 12 times tape stripping)	[65]
								(R)	*Amount of drug in SC strips (μg/cm^2^)* L_1.5_ S_0.5_ P_2_A 39.85 ± 1.29 *Control* L_2_ P80_2_A 28.32 ± 4.39	
Lornoxicam	L	Nanoemulsion NE8 Labrafac^®^ Tween 80 Pluronic F-68^®^ S_mix_ 3:1 Oil: S_mix_ 2:8 Nanoemulsion gel NG8 NE 8 gelled with Carbopol 934^®^ 1% Drug load (DL): 1.5%	Spontaneous aqueous phase titration	139 ± 29		0.233	23.87 ± 1.86	(M)	Full thickness pig abdominal skin Receptor: PBS (pH 7.4)	[79]
								(R)	*Flux J (μg·cm^−2^·h^−1^) in 24 h* NE8: 254.90 ± 1.25 NG8: 199.60 ± 6.93 *Control* (gel) 43.67 ± 2.11	
Indomethacin	L	Nanoemulsion F6 (O/W) Labrafil^®^ 5% Water 50% Tween 80 33.75% Transcutol-HP^®^ 11.25% S_mix_ ratio 3:1 S_mix_/oil ratio 4.00 Nanoemulsion gel NG6 F6 gelled with Carbopol 940^®^ 1% Triethanolamine 0.5% Drug load (DL): 0.5%	Spontaneous aqueous phase titration	F6 25.53 ± 2.22		F6 0.087	F6 14.32 ± 1.12	(M)	Full thickness rat abdominal skin Receptor: methanol-PBS (pH 7.4) (1:9)	[67]
								(R)	*Flux J (μg·cm^−2^·h^−1^)* F6: 73.96 ± 2.89 NG6: 61.64 ± 2.38 *Control* Indobene gel (Indo Gel™) 9.38 ± 0.41 *ER* F6: 7.88 NG6: 6.57	
Naproxen and Caffeine	L, H	Nanoemulsions with penetration enhancers in oil phase E1 Eucalyptol (EU) 15.93% Water 30.97% Volpo-N10^®^ 26.55% Ethanol 26.55% E2 Eucalyptol (EU) 14.63% Water 36.59% Volpo-N10^®^ 24.39% Ethanol 24.39% O1 Oleic acid (OA) 15.93% Water 30.97% Volpo-N10^®^ 26.55% Ethanol 26.55% O2 Oleic acid (OA) 14.63% Water 36.59% Volpo-N10^®^ 24.39% Ethanol 24.39% Drug load (DL): Caffeine 3% Naproxen 2% *Controls:* C1^C^: water 100% C2^C,N^: water 40%, ethanol 60% C3^C^: water 75%, PEG-6000 25% C4^N^: water 50%, ethanol 25%, Volpo-N10 25%	Spontaneous aqueous phase titration and moderate agitation	Caffeine E1: 19.3 ± 4.0 E2: 16.0 ± 3.6 O1: 5.9 ± 2.4 O2: 1.2 ± 0.1 Naproxen E1: 37.8 ± 5.9 E2: 25.0 ± 3.0 O1: 11.6 ± 3.8 O2: 13.5 ± 4.5	Caffeine/Naproxen-EU 15.3 Caffeine/Naproxen-OA 15.3		Caffeine/Naproxen-EU 13.7 ± 4.5 15.1 ± 4.0 Caffeine/Naproxen-OA 23.7 ± 4.7 28.3 ± 4.5	(M)	Full thickness human skin Receptor: PBS (pH 7.4)	[73]
								(R)	*Flux J (μg·cm^−2^·h^−1^) in 24 h* (Caffeine) E1: 263.6 ± 1.2 E2: 267.7 ± 24.0 O1: 118.8 ± 57.3 O2: 136.4 ± 95.2 *Control* (Caffeine) C1: 2.2 ± 0.8 C2: 25.6 ± 3.1 C3: 2.5 ± 0.7 C4: not identified *Flux J* (Naproxen) E1: 122.4 ± 27.1 E2: 86.6 ± 8.9 O1: 101.2 ± 41.7 O2: 74.0 ± 2.3 *Control* (Naproxen) C1: not identified C2: 23.4 ± 4.8 C3: 6.2 ± 0.3 C4: 7.3 ± 2.7	
Diclofenac diethylamine (DDEA)	L	Nanoemulsion F1 Oleic acid 15% Water 30% Polysorbate 20 18.3% Ethanol 36.7% S_mix_ 1:2 55% Nanoemulsion gel NE F1 gelled with Carbopol 971P^®^ 0.75% added Propylene glycol 10.0% Methyl paraben 0.18% Propyl paraben 0.02% Drug load (DL): 1.16% w/w DDEA (equivalent to 1% *w*/*w* diclofenac)	Spontaneous aqueous phase titration and vortex mixing	59.97 ± 3.22		0.28 ± 0.07	1.002	(M)	Strat-M^®^ membrane Receptor: PBS (pH 7.4): methanol (70:30)	[63]
								(R)	*Flux J (μg·cm^−2^·h^−1^) in 12 h* F1: 11.5 NE gel: 12.0 *Controls* DDEA solution: 1.71 Conventional gel: 11.7 Emulgel: 12.5 (coarse emulsion gel)	
Indomethacin	L	Nanoemulsion Triacetin^®^ Capryol 90^®^ 1:1 10% Water 40% Tween 80 25% Transcutol 25% Drug load (DL): 1%	Spontaneous aqueous phase titration and vortex mixing	101.1	n.a	n.a	60 ± 2.1	(M)	Full thickness hairless new born albino rat Receptor: PBS (pH 7.4)	[90]
								(R)	*Flux J (μg·cm^−2^·h^−1^) in 6 h* 55.81 ± 4.65 No control	
Meloxicam (MLX)	L	Nanoemulsion gel Caprylic acid 0.95% Water 70%Tween 80 20% Propylene glycol 10% Carbopol 940^®^ 0.05%	Spontaneous aqueous phase titration	125 ± 1.9	−31.85 ± 0.61	0.193 ± 0.01		(M)	Abdominal rat skin Receptor: Acetate buffer (pH 6.0)	[80]
								(R)	*Flux J (μg·cm^−2^·h^−1^)* 6.407 ± 0.0911 Control (MLX solution): not identified *Amount in skin layers in 24 h* Tape strips: SC level *Control* > MLX-NE gel (1.02 folds) Epidermal level MLX-NE gel > *Control* (3.24 folds) Dermal level MLX-NE gel > *Control* (1.42 folds)	
Flufenamic acid	L	Nanoemulsion Potassium sorbate 0.1% γ-Cyclodextrin 1.0% Water to 100% PCL-liquid (cetearyl ethyl hexanoate, isopropyl myristate) 20% Sucrose stearate S-970 2.5% Drug load (DL): 1%	High pressure homogenization	-	-	-	-	(M)	Dermatomed pig abdominal skin (1.2 mm) Receptor: PBS (pH 7.4)	[91]
								(R)	*Flux J (μg·cm^−2^·h^−1^)* γ–SN Fluf 1.83 ± 0.87 No control	
ANTIFUNGAL AGENTS										
Amphotericin B	L	Nanoemulsions F I Sefsol 218^®^ + DMSO 1:1 18.7% Water 44% Tween 80 Propylene glycol S_mix_ 2:1 37.3% F III Sefsol 218^®^ + DMSO 1:1 6% Water 64% Tween 80 Propylene glycol S_mix_ 1:2 30% F VI Sefsol 218^®^ + DMSO 1:1 16.8% Water 49.5% Tween 80 Propylene glycol S_mix_ 1:3 33.6% Drug load (DL): 0.1%	Spontaneous aqueous phase titration	FI 67.33 ± 0.8 F III 252 ± 1.0 F VI 74.2 ± 1.2	FI −37.305 F III −28.202 F VI −18.148	FI 0.635 F III 0.468 F VI 0.453	FI 25.4 ± 1 F III 40.7 ± 1.3 F VI 43.1 ± 1.4	(M)	Albino Wistar rat abdominal skin Receptor: 2% DMSO in PBS (pH 7.4)	[92]
								(R)	*Flux J (μg·cm^−2^·h^−1^)* F I: 18.02 ± 4.34 F III: 8.808 ± 3.55 F VI: 17.581 ± 2.56 *Controls* Drug solution 0.1% 5.895 ± 2.06 Fungisome® gel 0.1% 9.704 ± 5.74	
Amphotericin B	L	Nanoemulsion NE (FV) Sefsol-218^®^ 10% Water 65% Tween 80 Transcutol^®^ S_mix_ 1:3 25% AmpB-NE gel FV gelled with Carbopol 980^®^ 1% 1:1 Drug load (DL): 0.1%	Spontaneous aqueous phase titration	FV 76.2 ± 1.4 AmpB-NE gel: 97.04 ± 7.4	FV −31.48 AmpB-NE gel −39.27 ± 0.25	FV 0.303 AmpB-NE gel: 0.19 ± 0.01	FV 39.01 ± 1.4 AmpB-NE gel: 892 ± 9.64	(M)	Albino rat abdominal skin Receptor: 2% DMSO in PBS (pH 7.4)	[58]
								(R)	*Flux J (μg·cm^−2^·h^−1^)* FV: 15.74 ± 0.4 AmpB-NE gel 18.09 ± 0.6 *Control* (AmpB solution) 4.59 ± 0.01 *ER* FV: 8.97 AmpB-NE gel 10.42	
Terbinafine (TER) Citral (CIT)	L L	Nanoemulsion (NE) CIT 4% Water 71% Cremophor EL-40^®^ 18% 1,2-propylene glycol 6% S_mix_ 3:1 NG1 NE gelled with Carbopol 934^®^ 1% 1:1 (NG2 and NG3 contain 2% and 3% Carbopol 934^®^, respectively, at the same ratio with NE) Drug load (DL) in NE TER 1% and CIT 4% (oil phase) Controls: TER-CIT in Conventional gels (1.5% Carbopol 934^®^)	Spontaneous aqueous phase titration	NE 15.53 ± 3.32 NG1 14.88 ± 3.11	NE −7.4 ± 1.8 NG1 −6.5 ± 2.3	NE 0.074 ± 0.009 NG1 0.084 ± 0.025		(M)	Guinea pig abdominal skin Receptor: PBS (pH 7.4)	[93]
								(R)	*Flux J (μg·cm^−2^·h^−1^)* (TER) NE: 11.30 ± 0.56 NG1: 11.50 ± 0.43 *Control:* 1.48 ± 0.34 *Flux J* (CIT) NE: 54.71 ± 1.34 NG1: 55.01 ± 1.67 *Control:* 10.55 ± 0.87 *Amount in stratum corneum in 12 h (μg·cm^−2^)* NE-TER: 1.65 ± 0.29 NG1-TER: 6.27 ± 1.03 *Control TER:* 5.63 ± 0.76 NE-CIT: 0.95 ± 0.52 NG1-CIT: 10.88 ± 5.80 *Control CIT:* 13.68 ± 1.91 *Amount in epidermis-dermis in 12 h (μg·cm^−2^)* NE-TER: 73.5 ± 8.23 NG1-TER: 75.25 ± 9.52 *Control TER:* 17.42 ± 5.63 NE-CIT: 210.71 ± 12.38 NG1-CIT: 214.64 ± 0.92 *Control CIT:* 39.47 ± 5.51	
Fluconazole	H	Lecithin based NE PCL-liquid (cetearyl ethyl hexanoate, isopropyl myristate) 20% Potassium sorbate 0.1% γ-Cyclodextrin 1.0% Water to 100% Lipoid E-80^®^ 2.5% Drug load (DL): 1%	High pressure homogenization	LN Fluc 156.87 ± 09.73 γ-LN Fluc 155.60 ± 07.96	LN Fluc −24.70 ± 3.41 γ-LN Fluc −22.50 ± 2.20	LN Fluc 0.05 ± 0.01 γ-LN Fluc 0.07 ± 0.02		(M)	Dermatomed pig abdominal skin (1.2mm) Receptor: PBS (pH 7.4)	[91]
								(R)	*Flux J (μg·cm^−2^·h^−1^)* LN Fluc: 109.55 ± 11.30 γ-LN Fluc: 93.63 ± 3.80 No control	
CORTICOSTEROIDS										
Fludrocortisone acetate	L	Lecithin based NE PCL-liquid (cetearyl ethyl hexanoate, isopropyl myristate) 20% Potassium sorbate 0.1% γ-Cyclodextrin 0.5% or 1.0% Water to 100% Lecithin E-80^®^ 2.5% Drug load (DL): 1%	High pressure homogenization	γ-0.5% NE 171.03 ± 0.32 γ-1% NE 169.73 ± 2.35	γ-0.5% NE −33.17 ± 0.75 γ-1% NE −31.73 ± 1.52	γ-0.5% NE 0.098 ± 0.042 γ-1% NE 0.033 ± 0.049		(M)	Dermatomed pig abdominal skin (1.2mm) Receptor: PBS (pH 7.4)	[94]
								(R)	*Flux J (μg·cm^−2^·h^−1^)in 24 h* Finite dose γ-1% NE 0.067 ± 0.047 *NE Control:* 0.008 ± 0.007 Infinite dose γ-1% NE 2.48 ± 0.68 *NE Control:* 0.09 ± 0.07 *ER* of γ-1% NE: finite dose 8.38 infinite dose 27.55 *Control:* NE without cyclodextrin Applied as finite (5mg/cm^2^) and infinite doses (500mg/cm^2^) No significant different in drug flux between γ-1% NE and γ-0.5% NE	
Fludrocortisone acetate (FA) Flumethasone pivalate (FP)	L	Nanoemulsion (positive charge) PCL-liquid (cetearyl ethyl hexanoate, isopropyl myristate) 20% Lipoid S-75^®^ 4% α tocopherol 1% Phytosphingosine (PS) 0.4% or 0.6% Water to 100% Sucrose laurate L-1695 1% *or* Tween 80 1% Drug load (DL): 1% FA NL: FA NE with sucrose laurate L-1695 FA NT: FA NE with tween 80 FP NL: FP NE with sucrose laurate L-1695 FP NT: FP NE with tween 80	High pressure homogenization	FA NL 161 ± 0.7 FA NL-0.4PS 215 ± 2.8 FA NL-0.6PS 254 ± 2.2 FA NT 170 ± 3.8 FA NT-0.4PS 216 ± 26.6 FA NT-0.6PS 170 ± 2.1	FA NL −6.2 ± 0.4 FA NL-0.4PS +46 ± 0.4 FA NL-0.6PS +48 ± 0.7 FA NT −55 ± 0.7 FA NT-0.4PS +45 ± 0.7 FA NT-0.6 PS +48 ± 1.1	FA NL 0.12–0.22 FA NL-0.4PS 0.22–0.25 FA NL-0.6 PS 0.06–0.1 FA NT 0.15–0.18 FA NT-0.4PS 0.13–0.18 FA NT-0.6 PS 0.10–0.14		(M)	Dermatomed pig abdominal skin (1 mm) Receptor: PBS (pH 7.4)	[95]
								(R)	*Flux J (μg·cm^−2^·h^−1^) in 48 h* FA NL 0.126 ± 0.027 FA NL-0.4PS 0.150 ± 0.010 FA NL-0.6 PS 0.189 ± 0.012 FA NT 0.263 ± 0.043 FA NT-0.4PS 0.353 ± 0.018 FA NT-0.6 PS 0.377 ± 0.038 FP NT 2.290 ± 0.313 FP NT-0.4PS 2.698 ± 0.117 FP NT-0.6 PS 3.073 ± 0.104 No control Flux increased with PS concentration; Tween 80 > sucrose laurate	
Prednicarbate (PC)	L	Positively charged NE (PCNE) Phytosphingosine (PS) 0.6% Lecithin E-80^®^, Tween 80 2% Ethanol 20% α tocopherol 0.03% Potassium sorbate 0.1% Negatively charged NE (NCNE) Myristic acid 1% was used to replace PS Drug load (DL): 0.25%	High pressure homogenization	PCNE: 157 NCNE: 136	PCNE: 50–60 NCNE: −(40–50)	0.05–0.1		(M)	Full thickness human skin Receptor: Ethanol-PBS (1:1) No PC detected in receptor in 24 h	[53,54]
								(R)	*Amount PC in skin in 24 h* PCNE: 18.4 ± 3.4 μg/mL NCNE : 11.7 ± 2.5 μg/mL No control Positive > negative charged NE	
Fludrocortisone acetate (FA)	L	Lecithin based NE PCL-liquid (cetearyl ethyl hexanoate, isopropyl myristate) 20% Lecithin E-80^®^ 2.5% Potassium sorbate 0.1% γ-Cyclodextrin 1.0% Water to 100% Drug load (DL): 1%	High pressure homogenization	γ-LN Flud 175.82 ± 00.47	γ-LN Flud −30.19 ± 4.12	γ-LN Flud 0.09 ± 0.04		(M)	Dermatomed pig abdominal skin (1.2mm thick) Receptor: PBS (pH 7.4)	[91]
								(R)	*Flux J (μg·cm^−2^·h^−1^)* (FA) γ-LN Flud: 4.53 ± 0.99 No control	
VITAMINS										
α tocopherol (vitamin E)	L	Hyaluronic acid-based NE (L6) Methylene oxide (O) Tween 80-Span 20 (S) HA-GMS solution (A) Mass ratio O:S:A 2:3:95 Drug load (DL): 0.1% HA-GMS is water soluble amphiphile from crosslinking esterification of hyaluronic acid and glycerol α-mono stearate (stearin)	Oil/water/surfactant emulsifying system and solvent evaporation	57.3 ± 0.2		0.260		(M)	Full thickness Wistar rat dorsal skin Receptor: PBS (pH 7.4)	[96]
								(R)	*Flux J (μg·cm^−2^·h^−1^) in 24 h* L6: 14.68 ± 4.13 *Control*: not detected *Control*: 0.1% vitamin E in ethanol solution	
α tocopherol (vitamin E) and Vitamin K1 (VK1)	L	Nanoemulsions α-tocopherol (α-TOC), VK1 10% Water 64% Tween 80 10% Ethanol 16% Drug load (DL): 3% or 5%	Spontaneous aqueous phase titration and Ultrasonic nebulization NE-neb-VK1 = ultrasonic nebulizer	NE-VK1 3% 254.8 ± 10.7 NE-neb-VK1 3% 259.4 ± 4.1 NE-VK1 5% 215.7 ± 2.3 NE-neb-VK1 5% 233.2 ± 0.2	NE-VK1 3% −14.89 ± 2.68 NEs-neb-VK1 3% −16.60 ± 1.01 NE-VK1 5% −14.14 ± 0.29 NE-neb-VK1 5% −15.4 ± 0.1	NE-VK1 3% 0.22 ± 0.05 NEs-neb-VK1 3% 0.19 ± 0.14 NE-VK1 5% 0.23 ± 0.02 NE-neb-VK1 5% 0.26 ± 0.02		(M)	Pig ear skin (thickness 1.7–2.3 mm) Receptor: PBS : Ethanol (7:3 *v*/*v*)	[56]
								(R)	*Amount in epidermis in 24 h (ng/mg)* NEs-VK1 3%: 46.7 NEs-neb-VK1 3%: 72.8 NEs-VK1 5%: 55.6 NEs-neb-VK1 5%: 51.4 *Amount in dermis in 24 h (ng/mg)* NEs-neb-VK1 3%: 27.9 NEs-neb-VK1 5%: 24.8 No control	
MISCELLANEOUS										
Thiocolchicoside (TCC) anti inflammatory, analgesic, muscle relaxant	H	Nanoemulsion C1 (W/O type) Linseed oil : Sefsol^®^ 1:1 35.44% Water 10.81% Span 80 40.53% Transcutol P^®^ 13.51% S_mix_ 3:1 C3 (W/O type) Linseed oil : Sefsol^®^ 1:1 35.19% Water 9.26% Span 80 41.67% Transcutol P^®^ 13.89% S_mix_ 3:1 Drug load (DL): 0.2%	Spontaneous aqueous phase titration	C1 117.73 ± 13.71 C3 131.43 ± 15.15		C1 0.285 C3 0.311	C1 61.12 ± 5.28 C3 65.75 ± 6.08	(M)	Full thickness weanling pig abdominal skin Receptor: PBS (pH 7.4)	[97]
								(R)	*Flux J (μg·cm^−2^·h^−1^) in 24 h* (TCC) C1: 30.63 ± 4.18 C3: 28.01 ± 3.41 *Control* (TCC aqueous solution) 5.99 ± 0.73 *ER* C1: 5.114 C3: 4.676 Type of NE did not influence *ER*	
Curcumin natural anti-inflammatory	L	Nanoemulsion NE gel Glyceryl monooleate (GMO) Water Cremophor RH40^®^ PEG 400 O:S:CoS 1:8:1 Water: oil phase 5:1 NE gelled with Viscolam AT 100P^®^ 5% and added: Methyl paraben 0.2% Propyl paraben 0.05% Glycerine 5% Propylene glycol 15% Drug load (DL): 0.35%	Spontaneous aqueous phase titration with 1 h ultrasonic sonication	85.0 ± 1.5	0.18 ± 0.0	−5.9 ± 0.3	2000–2700	(M)	Shed snake skin Receptor: PBS (pH 7.4)	[91]
								(R)	*Flux J (μg·cm^−2^·h^−1^)* NE gel: 1.699 ± 0.050 *Control gel* 0.836 ± 0.004	
Bovine albumin-fluorescein isothiocyanate conjugate (FITC-BSA) vaccine model	L	Nanoemulsion Squalene 37.5% Water 52.5% Span 80, Tween 80 10% S_mix_ 1:1 Drug load (DL): 0.25%	Spontaneous aqueous phase titration with high pressure homogenization	85.2 ± 15.5	−45.17 ± 4.77	0.186 ± 0.026	14.6 ± 0.026	(M)	Mouse skin Receptor: PBS (pH 7.4)	[69]
								(R)	*Flux J (μg·cm^−2^·h^−1^) in 48 h* NE: 23.44 ± 17.230 *Controls* CE: 6.10 ± 0.977 CA: 3.15 ± 0.897 *Controls* CE: emulsifiers solution (10% of S_mix_) CA: aqueous solution	
Granisetron HCl (GHCl) anti emetic drug	H	Nanoemulsion with penetration enhancer NMP Isopropyl myristate (IPM) 4% Tween 85 20% Ethanol 20% N-methyl pyrrolidone (NMP) 10% Water up to 100% Drug load (DL): 2.5%	Spontaneous aqueous phase titration	48.3 ± 1.7		0.27 ± 0.02		(M)	Full thickness rat abdominal skin Receptor: saline solution	[98]
								(R)	*Flux J (μg·cm^−2^·h^−1^)* NMP NE: 85.39 ± 2.90 *Control*: 71.17 ± 3.54 *Amount in skin in 12 h (μg·cm^−2^)* NMP NE: 891.8 ± 2.86 *Control*: 889.1 ± 2.24 NMP NE ≅ NE Control: NE without NMP	
Minoxidil (Min) antihypertensive vasodilator (stimulate hair growth)	H	Lecithin based NE PCL-liquid (cetearyl ethyl hexanoate, isopropyl myristate) 20% Potassium sorbate 0.1% γ-Cyclodextrin 1.0% Water to 100% Lecithin E-80^®^ 2.5% Drug load (DL): 1%	High pressure homogenization	-	-	-		(M)	Dermatomed pig abdominal skin (1.2mm thick) Receptor: PBS (pH 7.4)	[91]
								(R)	*Flux J (μg·cm^−2^·h^−1^)* 102.56 ± 9.41 No control	
